# Beyond Anti-viral Effects of Chloroquine/Hydroxychloroquine

**DOI:** 10.3389/fimmu.2020.01409

**Published:** 2020-07-02

**Authors:** Vincent Gies, Nassima Bekaddour, Yannick Dieudonné, Aurélien Guffroy, Quentin Frenger, Frédéric Gros, Mathieu Paul Rodero, Jean-Philippe Herbeuval, Anne-Sophie Korganow

**Affiliations:** ^1^Université de Strasbourg, INSERM UMR - S1109, Strasbourg, France; ^2^Hôpitaux Universitaires de Strasbourg, Department of Clinical Immunology and Internal Medicine, National Reference Center for Systemic Autoimmune Diseases (CNR RESO), Tertiary Center for Primary Immunodeficiencies, Strasbourg, France; ^3^Université de Strasbourg, Faculty of Pharmacy, Illkirch, France; ^4^Université de Paris, CNRS UMR-8601, Paris, France; ^5^Team Chemistry & Biology, Modeling & Immunology for Therapy, CBMIT, Paris, France; ^6^Université de Strasbourg, Faculty of Medicine, Strasbourg, France; ^7^Université de Strasbourg, Faculty of Life Sciences, Strasbourg, France

**Keywords:** chloroquine, hydroxychloroquine, COVID-19, SARS-CoV2, interferon, TLR, STING, RIG-I

## Abstract

As the world is severely affected by COVID-19 pandemic, the use of chloroquine and hydroxychloroquine in prevention or for the treatment of patients is allowed in multiple countries but remained at the center of much controversy in recent days. This review describes the properties of chloroquine and hydroxychloroquine, and highlights not only their anti-viral effects but also their important immune-modulatory properties and their well-known use in autoimmune diseases, including systemic lupus and arthritis. Chloroquine appears to inhibit *in vitro* SARS virus' replication and to interfere with SARS-CoV2 receptor (ACE2). Chloroquine and hydroxychloroquine impede lysosomal activity and autophagy, leading to a decrease of antigen processing and presentation. They are also known to interfere with endosomal Toll-like receptors signaling and cytosolic sensors of nucleic acids, which result in a decreased cellular activation and thereby a lower type I interferons and inflammatory cytokine secretion. Given the antiviral and anti-inflammatory properties of chloroquine and hydroxychloroquine, there is a rational to use them against SARS-CoV2 infection. However, the anti-interferon properties of these molecules might be detrimental, and impaired host immune responses against the virus. This duality could explain the discrepancy with the recently published studies on CQ/HCQ treatment efficacy in COVID-19 patients. Moreover, although these treatments could be an interesting potential strategy to limit progression toward uncontrolled inflammation, they do not appear *per se* sufficiently potent to control the whole inflammatory process in COVID-19, and more targeted and/or potent therapies should be required at least in add-on.

## Introduction

Chloroquine (CQ) and hydroxychloroquine (HCQ) are “old” drugs but are still widely used in very diverse situations, including infectious diseases (1–3), rheumatic/inflammatory diseases (4), or in clinical research protocols as add-on cancer therapy (5). Indeed, they are cheap and safe considering rare effective ocular toxicity (<2%) and acute cardiac toxicity. However, clinicians should be warned that CQ/HCQ treatments, especially with high doses, can be complicated with heart failure, or non-reversible conduction disorders (6).

As the world is severely affected by COVID-19 pandemic, the use of CQ/HCQ in prevention or for the treatment of patients is at the center of much controversy in recent days. We thus believe appropriate to make a short review and discuss about their pharmacological properties and the ways CQ/HCQ are able to interfere with innate or adaptative immune system.

## History of HCQ/CQ

HCQ and CQ are weak bases with a common flat aromatic core structure. These decades-old drugs are in fact synthetic antimalarial drugs, although HCQ is above all a major treatment for systemic lupus (7). The story of antimalarials starts with the cinchona bark that was already used by the Incas for its antipyretic property rather than malaria treatment itself. It was only in 1820, that Pelletier and Caventou, french pharmacists, isolated the fundamental antimalarial alkaloid: quinine. During world war II, American soldiers who fought in the pacific region received antimalarials (quinacrine) in prophylaxis, showing a beneficial effect of this compound on lupus and rheumatoid arthritis. CQ was subsequently introduced in 1943, showing its beneficial effect in systemic lupus erythematosus in 1953 (8). CQ cardiac and retinal side effects led to the development, in 1955, of a hydroxylated derivative: HCQ, a little less active but above all less toxic molecule (9, 10). HCQ holds actually a major place in the treatment of autoimmune/ inflammatory rheumatic or dermatological diseases.

## Lysosomal Activity and Autophagy

Autophagy is a catabolic homeostatic or induced process that involves the sequestration of cytoplasmic components in double-membraned autophagosomes. Autophagosomes ultimately fuse with lysosomes leading to the degradation of their content. Autophagy is a major player in immunity (11). It contributes to reduce inflammation by modulating type I interferon production and inflammasome activity. Autophagy is a countermeasure to infectious diseases clearing intracellular pathogens but autophagic membrane can conversely constitute hubs for viral replication. Autophagy also contributes to generate antigens processed on MHC class II (12).

**Figure 1:**
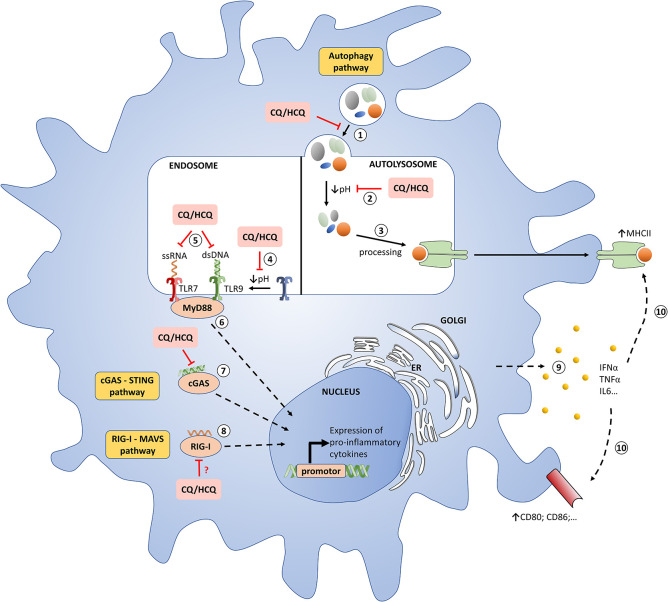
Molecular mechanisms of chloroquine/hydroxychloroquine in pDCs. **(1)** Autophagosome-lysosome fusion. CQ/HCQ impair this step. **(2)** Degradation of cargo from autophagosome. CQ/HCQ accumulate in lysosomes (lysosomotropism) and inhibit their function by increasing the pH. **(3)** Antigens are processed on MHC class II. **(4)** Proteolysis of TLRs by acid-dependent proteases is an essential step for the recognition of ligands. CQ/HCQ inhibit acid-dependent proteases by increasing the pH. **(5)** TLRs interact with nucleic acids presented to endosomal compartments. HCQ and CQ can bind directly to nucleic acids, preventing their recognition and inhibiting TLR-ligand interactions. **(6)** TLRs activation lead to MyD88 recrutement with a subsequent synthesis of pro-inflammatory cytokines, especially IFN-I. **(7)** Cytosolic DNA binds to cGAS, which then synthesizes the second messenger cGAMP to mediate STING-dependent transcription of IFN-I. HCQ and CQ block the binding of dsDNA / cGAS, thus attenuating the underlying activation of the STING pathway mediated by cGAMP. **(8)** Cytosolic RNA is recognized by RIG-I, the signal is transferred to MAVS, then to subsequent interactors leading to expression of IFN-I. CQ/HCQ may impair this process. (**9** and **10**) The release of IFNα, among other cytokines, stimulates a feedback activation with notably MHCII and co-stimulatory molecules upregulation. CQ: chloroquine; dsDNA, double-stranded DNA; ER, endoplasmic reticulum; HCQ: hydroxychlroquine; IFN-I: type I interferons; MHC: major histocompatibily complex; pDC: plasmacytoid dendritic cell; TLR : toll like receptor.

An important mode of action of HCQ is the inhibition of lysosomal activity (Figure 1 and Table 1). As weak bases (Figure S1A), CQ and HCQ accumulate in lysosomes (lysosomotropism) and inhibit their function. *In vitro*, CQ can disrupt the endolysosomal system and therefore destabilize the lysosomal membranes leading to intracellular release of lysosomal enzymes and impairment of autophagosome–lysosome fusion (13, 14). As a consequence LC3-II (microtubule-associated protein light chain 3-II), a lipidated protein normally associated with autophagosomes until degradation/recycling after fusion with lysosomes, accumulates under HCQ incubation (Figure S1B) (13, 25). Lysosomes are involved not only in the recycling of cellular substrates but also in the processing of antigens and their presentation on MHC class II proteins (4, 26–29). Thus, CQ/HCQ decrease, within 1 h *in vitro*, antigen processing and presentation by antigen presenting cells (4, 15, 16). Other mechanisms are invoked for the impact of HCQ or CQ on autophagy. Recently Rebecca et al. identified that inhibition of palmitoyl-protein thioesterase 1 (PPT1) is, at least in part, responsible for the observed anti-autophagy effect (30).

**Table 1:** Main mechanisms of actions of CQ/HCQ.

**Mode of actions**	**Molecular mechanisms**	**References**
Impairment of lysosomal activity and autophagic process	CQ/HCQ accumulate in lysosomes (lysosomotropism) and inhibit their functions	(4, 13–16)
	CQ/HCQ Impair of autophagosome–lysosome fusion	
	CQ/HCQ decrease antigen processing and presentation, by antigen presenting cells	
Interference with TLR signaling	Accumulation of CQ/HCQ in lysosomes raises endosomal pH and hinders the signaling of TLR 3,7, and 9	(17–21)
	Direct binding of CQ/HCQ to nucleic acids prevents their recognition and inhibits TLR-ligand interactions	(18, 22)
Inhibition of cytosolic sensors of nucleic acids	CQ/HCQ reduce activation of STING pathway: CQ/HCQ block the binding of dsDNA to cGAS.	(23, 24)
	CQ/HCQ reduce activation of RIG-I, pathway? *(further investigations required)*	(24)

*CQ, chloroquine; dsDNA, double-stranded DNA; HCQ, hydroxychlroquine*.

## Toll Like Receptors

Perhaps the most important advance in our understanding of CQ/HCQ has been the discovery of their inhibiting effects on toll like receptors (TLRs), one of our first line of defense against bacterial and viral agents. HCQ and CQ inhibit some endosomal TLRs, mostly TLR7 and TLR9, which are able to recognize viral, bacterial and endogenous nucleic acids (Figure 1 and Table 1) (4, 31). These TLRs are located in the intracellular compartments to minimize accidental exposure to self-nucleic material (31). TLR7 is activated by ssRNA compounds, while TLR9 is activated by unmethylated CpG DNA (32). Activation of these endosomal TLRs can significantly contribute to promoting inflammation and/or the development of autoimmune diseases, such as systemic lupus erythematosus (SLE) (33, 34). Therefore, inhibition of endosomal TLRs, and consequently type I interferons (IFN-I) production, holds great therapeutic potential for the treatment of autoimmune diseases (35). CQ/HCQ have shown their ability to inhibit, in mice and *in vitro*, the TLR9 stimulation induced by CpG and the underlying production of IL-6 and TNFα (36, 37). These drugs can also inhibits RNA-mediated activation of TLR7 signaling and the production of IFNα (38, 39). In addition to cytokines secretion, TLR7 and 9 inhibition will also impair costimulatory molecules expression, such as CD86 on B cells, contrary to stimulation with pokeweed mitogen (Figure S1C) (40). Two modes of action are currently suggested:

- Raising endosomal pH has been proposed as the explanation for defective maturation and antigen presentation as well as impaired responses to TLR activation. Indeed, acidic conditions are necessary for strong interactions of nucleic acids with TLR9 (17). In this way, blocking endosomal acidification hinders the signaling of TLR 3, 7, and 9 (18–20). Ewald et al. showed that cleavage of TLR9 does not take place in the absence of acidification of the endolysosomal compartments. However, the proteolysis of TLR9 is an essential step for the recognition of ligands (CpG for TLR9) and the recruitment of MyD88, and consequently the signaling of TLR9 (21).
- CQ/HCQ can also directly bind to nucleic acids and such interferences lead to structural changes in the TLR ligands, preventing their recognition and inhibiting TLR-ligand interactions (18, 22).

## Plasmacytoid Dendritic Cells

The capacity of plasmacytoid dendritic cells (pDCs) to produce massive quantities of type I IFN has driven our understanding about the biology of these cells and their major role in both immunity against virus and in inflammation/autoimmunity (41).

pDCs can be acutely activated through different cell surface receptors and cytosolic sensors but TLR7/9 are likely their dominant mode of activation for endogenous or exogenous nucleic acids, with respect to IFN-I production (41–45). When triggered, pDCs expressed costimulatory molecules, and are able to prime subsequently T cells. Within 10 h following the sensing of nucleic acids, more than 80% of genes expressed in pDCs are IFNs or driven by IFNs (41, 46, 47). In addition, pDCs secrete cytokines as IL-6, TNFα and IL-12 and control the expression of many inflammatory cytokines. Because of these properties, they are considered as one of the main cellular actor of anti-viral defense (45).

In parallel, pDCs and IFN-I, have been largely studied in chronic inflammatory/autoimmune diseases and systemic lupus (SLE) (48). IFN-I are involved in SLE physiopathology through multiple mechanisms, focusing recent therapeutic research and trials (49, 50). HCQ is essential in the treatment of the disease and in prevention of flares, and CQ/HCQ are able to decrease IFNs production by pDCs (Figure S1D) (22, 51). In addition, HCQ/CQ effects are not limited to pDCs and result in several other cytokines inhibition (Figures S1E,F) (22, 52–54).

## Cytosolic Sensors of Nucleic Acids

Apart from TLRs, HCQ/CQ interfere with other pattern recognition receptors (PRRs) essential to the anti-viral response namely the cGAS–STING and RIG-I–MAVS pathways (Figure 1 and Table 1). These cytosolic PRRs recognize and respond to DNA and RNA, respectively (23, 55). Cytosolic DNA is recognized by the cGAS-STING signaling axis which stimulates antiviral immunity by inducing IFN-I (56, 57). More precisely, cytosolic DNA binds to cGAS, which then synthesizes the second messenger cGAMP to mediate STING-dependent transcription of IFN-I (57). CQ/HCQ block the binding of dsDNA to cGAS, thus attenuating the underlying activation of the STING pathway mediated by cGAMP (23, 24).

Cytosolic RNA is recognized by RIG-I, the signal is transferred to MAVS, then to subsequent interactors leading to expression of IFN-I (55). An et al. showed that CQ/HCQ may inhibit RIG-I–stimulated induction of IFNs (24). However, the potential impacts of CQ/HCQ on this pathway need further investigations.

## Other Effects Of CQ/HCQ

Considering CQ/HCQ and pathogens direct interactions, only the interference of CQ and *Plasmodium*, via hemozoin crystallization inhibition leading to parasite death, has been clearly documented (58). Immunity against infectious agents, including bacteria, virus or fungus, also involve vessels, and inflammation at the endothelium level (59). In this view, CQ/HCQ has been suggested to prevent prothrombotic state and endothelial dysfunction (60, 61). Finally, neutrophil extracellular traps (NETs) and the ability to mount NETosis is essential in innate immunity against pathogens. However, CQ is also effective as an early upstream inhibitor of NET formation in murine models of inflammation (62).

## CQ/HCQ and SARS-CoV2 Infection

Considering SARS-CoV2, which is at the center of current concerns, some studies argue for lower nasopharyngeal carriage after 6 days (63) and an overall improvement of patients with CQ/HCQ (63–66), while more recent studies do not show rapid viral clearance or lower mortality (67–73).

Little is known about the mechanisms of action of CQ/HCQ during SARS-CoV2 infection. CQ has repeatedly demonstrated its ability, *in vitro* or in mouse models, to inhibit the replication of various strains of coronavirus, including those responsible for severe acute respiratory syndrome (SARS) in 2002 and 2003 (74–76). Additionally, *in vitro* studies carried out on SARS-CoV2 responsible for COVID-19 infection also confirm the potential interest of CQ/HCQ (77–79). Indeed, the increased endosomal pH due to CQ/HCQ may affect early stage of viral replication, via inhibition of virus-endosome fusion (80), and seem to stall the release of viral genome (77, 79). Although a direct interaction between HCQ/CQ and the virus has yet not been described, *in vitro* CQ interferes with terminal glycosylation of the SARS coronavirus receptor ACE2 (75, 81). Strikingly *ACE2* is an interferon stimulated gene (82, 83), as CQ/HCQ decrease IFN-I secretion, they may also hinder the expression of the viral receptor in the neighboring airway epithelial cells (83).

## Discussion

Thanks to its known capacity to inhibit both TLRs and cGAS-STING pathway, HCQ is a first line treatment in SLE and other autoimmune/inflammatory conditions. More than 70% of SLE patients around the world are under HCQ therapy which represents today indeed thousands of people. From its wide use, we know that HCQ efficiency takes a few weeks to impact clinical symptoms, as arthritis, or cutaneous manifestations (7). However, despite the first use of CQ and HCQ in immune related disease almost 70 years ago, their exact mechanisms of action are only beginning to be understood, whether considering pharmacokinetic or molecular mechanisms. In any case, *in vitro* CQ/HCQ inhibit the production of IFN-I and inflammatory cytokines by innate immune cells. However, considering cytokines profile in SLE patients under diverse immunosuppressive therapies compared to untreated patients (84), HCQ *per se* is far from being the most efficient.

Recently, a new coronavirus SARS-CoV2 infection (or COVID-19) has started to spread around world with the development of pneumonia and severe acute respiratory syndrome (SARS). CQ/HCQ remain at the center of a therapy controversy as several publications describe an improvement of patients with CQ/HCQ (63–66), while others give results showing rather their ineffectiveness (67–73). Undoubtedly, several mechanistical arguments are in favor of HCQ/CQ interfering with coronavirus infection and virus spreading. However, we know that COVID-19 is not only an acute viral infection. Some patients experience a biphasic evolution/two-step disease progression. They are first infected, viremic, with flu-symptoms. At this stage, they may develop mild respiratory manifestations. Some days later, for yet unclear reasons, disease can dramatically worsen with an inflammatory procession leading to a subsequent “cytokine storm,” a pro-thrombotic state and even a macrophage activation syndrome with heavy lung damage and/or multiorgan failure (85–87).

Looking back to SARS-CoV models, it has been suggested and documented in mice that the first phase of the disease could be accompanied by an initial virus mediated and adapted IFN-I response. The second phase of the disease would occur secondary to an inappropriate delayed IFN-I production in infected lungs. This leads to a subsequent and excessive innate immune response with pathogenic inflammatory monocyte-macrophages and sub-optimal T cell response, partly due to T cell apoptosis, which is not enough to dampen immune innate system overactivation (88). Accordingly, a recent study from Hadjadj et al. identified an impaired IFN-I activity, increased T cell apoptosis and exacerbated inflammatory responses in severe COVID-19 patients (89). Thus, like what has been suggested for SARS-CoV-1 infection (88), the driving clinical features of severe COVID-19 patients stem from a dysregulated immune response in patients with notably a delayed and abnormal production of IFN-I (82, 89).

Hence, what are our aims when we treat SARS-CoV2 infection with CQ/HCQ ? Do we wish to decrease a beginning viremia by endolysosomal pathway inhibition with CQ/HCQ, which may further delay the anti-viral IFN-I response? or do we wish to control the inflammatory storm by interfering with PRRs and endosomal TLRs signaling, leading to a decreased cellular activation and thereby cytokine secretion? Then, if we initiate the treatment in the second phase of the disease, when the disease becomes somehow an inflammatory disease, are we really in frame with CQ/HCQ already demonstrated properties?

It appears from our clinical experience and from national or international series of COVID19 infected patients, that men are more affected than women (69, 90) and patients with autoimmune diseases, including SLE who are under HCQ or other immunosuppressive drugs, are not drastically affected. This could have other explanations than HCQ efficiency: (i) most of patients with autoimmunity are women, (ii) some of them present an intrinsic IFN-I response which is not completely abolished by HCQ and remains more intense than healthy donors, (iii) immunosuppressive therapies could impact or prevent high auto-inflammatory conditions.

At this time, we do not have any definitive clue concerning CQ/HCQ impacts on the first anti-viral phase of COVID-19, and the use of CQ/HCQ must remain cautious as results from recent studies do not support these treatments (67, 69, 72). Considering published data on COVID-19 disease and older data about SARS physiopathology, and although CQ/HCQ remain an interesting potential strategy to limit progression toward uncontrolled inflammation, they do not appear *per se* to be sufficient to control the whole inflammatory process in COVID-19, and more targeted and/or potent therapies should be required at least in add-on.

## Author Contributions

VG, AG, MR, J-PH, and A-SK wrote the manuscript. VG, AG, YD, FG, J-PH, and A-SK performed the literature search. VG, YD, and NB designed the figures. NB, VG, AG, YD, and QF performed the experiments. NB, VG, YD, AG, QF, FG, MR, J-PH, and A-SK interpreted the data. All authors reviewed and approved the manuscript.

## Conflict of Interest

AG received funding from Shire Takeda, outside the submitted work. The remaining authors declare that the research was conducted in the absence of any commercial or financial relationships that could be construed as a potential conflict of interest.

## Abbreviations

CQ: chloroquine
HCQ: hydroxychloroquine
IFN: interferon
IFN-I: type I interferons
NET: neutrophil extracellular trap
pDC: plasmacytoid dendritic cell
SARS: severe acute respiratory syndrome
SLE: systemic lupus erythematosus
TLR: toll like receptor.
