# Supplementary material for: Beyond Anti-viral Effects of Chloroquine/Hydroxychloroquine
Source: Front Immunol. 2020 Jul 2;11:1409. doi: 10.3389/fimmu.2020.01409 (PMC7343769; doi:10.3389/fimmu.2020.01409)
Supplement: Supplementary file 1 [file Data_Sheet_1.docx]

**BEYOND ANTI-VIRAL EFFECTS OF CHLOROQUINE/HYDROXYCHLOROQUINE**

**Section: MINI REVIEW**

**Supplementary materials**

Vincent Gies^1,2,3^ (PharmD, PhD), Nassima Bekaddour^4,5^ (MSc), Yannick Dieudonné^1,2,6^ (MD), Aurélien Guffroy^1,2,6^ (MD, PhD), Quentin Frenger^1,7^ (MSc), Frédéric Gros^1,7^ (PhD), Mathieu Rodero^4,5^ (PhD), Jean-Philippe Herbeuval^4,5^* (PhD), Anne-Sophie Korganow^1,2,6^* (MD, PhD)

^1^Université de Strasbourg, INSERM UMR - S1109, F-67000 Strasbourg, France.

^2^Hôpitaux Universitaires de Strasbourg, Department of Clinical Immunology and Internal Medicine, National Reference Center for Systemic Autoimmune Diseases (CNR RESO), Tertiary Center for Primary Immunodeficiencies, F-67000 Strasbourg, France.

^3^Université de Strasbourg, Faculty of Pharmacy, F-67400 Illkirch, France.

^4^Université de Paris, CNRS UMR-8601, F-75006 Paris, France.

^5^Team Chemistry & Biology, Modeling & Immunology for Therapy, CBMIT, Paris, France.

^6^Université de Strasbourg, Faculty of Medicine, F-67000 Strasbourg, France.

^7^Université de Strasbourg, Faculty of Life Sciences, F-67000 Strasbourg, France.

*These authors contribute equally to the work

**Correspondence:**

Jean-Philippe Herbeuval, PhD

Chemistry & Biology, Modeling & Immunology for Therapy

CNRS UMR-8601, Faculté des Saints Pères Université de Paris

45 rue des Saints-Pères, 75006 Paris, FRANCE

Phone: + 33 01 42 86 38 32 Fax : 01 42 86 43 84

E-mail: [jean-philippe.Herbeuval@parisdescartes.fr](javascript:;)

Prof. Anne-Sophie Korganow, MD, PhD

Nouvel Hôpital Civil, INSERM UMRS-1109

1 place de l’Hôpital, 67000 Strasbourg, FRANCE

Phone: + 33 3 88 41 70 25; Fax: + 33 3 88 61 06 80

E-mail: [korganow@unistra.fr](mailto:korganow@unistra.fr)

**FIGURE**

**FIGURE S1. CQ and HCQ inhibit *in vitro* autophagy, TLR-dependent cellular activation and cytokine production.** **(A)** CQ and HCQ are synthetic antimalarial drugs and weak bases with a common flat aromatic core structure. **(B)** CQ/HCQ impair autophagosome–lysosome fusion, consequently LC3-II accumulates under CQ/HCQ incubation (1–3). Histograms represent LC3 molecules MFI in monocytes or T cells from PBMCs of HDs, in the presence or absence of HCQ (60µM), after *in vitro* incubation for 2 hours. **(C)** CQ/HCQ inhibit endosomal TLR activation and impair costimulatory molecules expression (4–6). Histograms represent CD86 molecules MFI on B cells from PBMCs of HD, in the presence or absence of HCQ (20µM), after no stimulation or *in vitro* stimulation with imiquimod (2µg/mL; TLR7 ligand), CpG (0,5µg/mL; TLR9 ligand) or pokeweed mitogen as control (2µg/mL) for 48 hours. **(D and E)** HCQ decreases IFNs production (7–9). Levels of IFNs (type I and II) were measured in culture supernatants from HDs PBMCs or pDCs, pretreated during 1 hour with increasing or fixed (20µM) concentration of CQ, then stimulated with R848 (5µg/mL; TLR7/8 agonist) for 24 hours. IFNs quantification was performed with the STING-37 reporter cell line. **(F)** CQ/HCQ incubation lead to several other cytokine secretion inhibition (7,9–12). PBMCs from HDs were preincubated or not during 1 hour with CQ (20µM), then stimulated with R848 (5µg/mL; TLR7/8 agonist) for 16 hours and analysed by mass cytometry. CQ: chloroquine; HCQ: hydroxychloroquine; HD: healthy donor; IFN: interferon; mDC: myeloid dendritic cell; MFI: median of fluorescence intensity; NS: non-stimulated; PBMC: peripheral blood mononuclear cells; pDC: plasmacytoid dendritic cell.

**BIBLIOGRAPHY**

1. Mauthe M, Orhon I, Rocchi C, Zhou X, Luhr M, Hijlkema K-J, Coppes RP, Engedal N, Mari M, Reggiori F. Chloroquine inhibits autophagic flux by decreasing autophagosome-lysosome fusion. *Autophagy* (2018) **14**:1435–1455. doi:10.1080/15548627.2018.1474314

2. Klionsky DJ, Abdelmohsen K, Abe A, Abedin MJ, Abeliovich H, Acevedo Arozena A, Adachi H, Adams CM, Adams PD, Adeli K, et al. Guidelines for the use and interpretation of assays for monitoring autophagy (3rd edition). *Autophagy* (2016) **12**:1–222. doi:10.1080/15548627.2015.1100356

3. Circu M, Cardelli J, Barr MP, O’Byrne K, Mills G, El-Osta H. Modulating lysosomal function through lysosome membrane permeabilization or autophagy suppression restores sensitivity to cisplatin in refractory non-small-cell lung cancer cells. *PLoS ONE* (2017) **12**:e0184922. doi:10.1371/journal.pone.0184922

4. Schrezenmeier E, Dörner T. Mechanisms of action of hydroxychloroquine and chloroquine: implications for rheumatology. *Nat Rev Rheumatol* (2020) **16**:155–166. doi:10.1038/s41584-020-0372-x

5. Wallace DJ, Gudsoorkar VS, Weisman MH, Venuturupalli SR. New insights into mechanisms of therapeutic effects of antimalarial agents in SLE. *Nat Rev Rheumatol* (2012) **8**:522–533. doi:10.1038/nrrheum.2012.106

6. Gies V, Schickel J-N, Jung S, Joublin A, Glauzy S, Knapp A-M, Soley A, Poindron V, Guffroy A, Choi J-Y, et al. Impaired TLR9 responses in B cells from patients with systemic lupus erythematosus. *JCI Insight* (2018) **3**: doi:10.1172/jci.insight.96795

7. Kužnik A, Benčina M, Švajger U, Jeras M, Rozman B, Jerala R. Mechanism of Endosomal TLR Inhibition by Antimalarial Drugs and Imidazoquinolines. *The Journal of Immunology* (2011) **186**:4794–4804. doi:10.4049/jimmunol.1000702

8. Sacre K, Criswell LA, McCune JM. Hydroxychloroquine is associated with impaired interferon-alpha and tumor necrosis factor-alpha production by plasmacytoid dendritic cells in systemic lupus erythematosus. *Arthritis Res Ther* (2012) **14**:R155. doi:10.1186/ar3895

9. Smith N, Rodero MP, Bekaddour N, Bondet V, Ruiz-Blanco YB, Harms M, Mayer B, Bader-Meunier B, Quartier P, Bodemer C, et al. Control of TLR7-mediated type I IFN signaling in pDCs through CXCR4 engagement—A new target for lupus treatment. *Science Advances* (2019) **5**:eaav9019. doi:10.1126/sciadv.aav9019

10. Picot S, Peyron F, Vuillez JP, Polack B, Ambroise-Thomas P. Chloroquine inhibits tumor necrosis factor production by human macrophages in vitro. *J Infect Dis* (1991) **164**:830. doi:10.1093/infdis/164.4.830

11. Jang C-H, Choi J-H, Byun M-S, Jue D-M. Chloroquine inhibits production of TNF-alpha, IL-1beta and IL-6 from lipopolysaccharide-stimulated human monocytes/macrophages by different modes. *Rheumatology (Oxford)* (2006) **45**:703–710. doi:10.1093/rheumatology/kei282

12. van den Borne BE, Dijkmans BA, de Rooij HH, le Cessie S, Verweij CL. Chloroquine and hydroxychloroquine equally affect tumor necrosis factor-alpha, interleukin 6, and interferon-gamma production by peripheral blood mononuclear cells. *J Rheumatol* (1997) **24**:55–60.
